# Reduced parenting stress following a prevention program decreases internalizing and externalizing symptoms in the offspring of parents with bipolar disorder

**DOI:** 10.1186/s40345-022-00284-2

**Published:** 2023-02-27

**Authors:** Tiffany Resendes, Lisa Serravalle, Vanessa Iacono, Mark A. Ellenbogen

**Affiliations:** Centre for Research in Human Development, Department of Psychology, Concordia University, 7141 Sherbrooke St. West, Montreal, QC H4B 1R6 Canada

**Keywords:** Preventative intervention, Bipolar disorders, Parenting stress, Internalizing and externalizing symptoms

## Abstract

**Background:**

Offspring of parents with bipolar disorder (OBD) are at risk for developing mental disorders, and the literature suggests that parenting stress may represent an important risk factor linking parental psychopathology to offspring psychopathology. We aimed to investigate whether improvements in parenting stress mediated the relationship between participation in a prevention program and offspring internalizing and externalizing symptoms at follow-up.

**Methods:**

Families having a parent with BD (*N* = 25) underwent a 12-week prevention program. Assessments were conducted at pre-intervention, post-intervention, and at 3- and 6-month follow-ups. Families of parents with no affective disorders (i.e., control families) served as a comparison sample (*N* = 28). The *Reducing Unwanted Stress in the Home* (RUSH) program aimed to teach communication, problem-solving, and organization skills to improve the rearing environment. Measures included the Parenting Stress Index—4th Edition, the Behaviour Assessment Scales for Children—2nd Edition, and the UCLA Life Stress Interview.

**Results:**

Families having a parent with BD reported more parenting stress at pre-intervention, and more change across time, than control families. Improvements in parenting stress mediated the relationship between participation in the intervention and reduced offspring internalizing and externalizing symptoms. While families having a parent with BD reported more chronic interpersonal stress at pre-intervention, no intervention effects were found.

**Conclusions:**

The findings demonstrate that a preventative intervention targeting parenting stress in families may serve to prevent the development of mental disorders in at-risk children.

## Background

Offspring of parents living with bipolar disorder (OBD) are at heightened risk for developing mental disorders (Duffy et al. 2014; Mesman et al. 2013; Nijjar et al. 2014). Despite evidence of BD’s heritability (McGuffin et al. 2003), environmental factors may have a robust influence on the OBD’s developmental outcomes (Ferreira et al. 2013; Stapp et al. 2020). Previous research indicates BD in parents might influence their offspring’s functioning through parenting practices and by creating a stressful family environment. Specifically, families having a parent with BD, relative to families with parents having no mental disorders, are known to have ineffective parenting practices, poor family cohesion, disorganization, poor marital adjustment, and dysfunctional parent–child interactions (Ferreira et al. 2013; Ellenbogen and Hodgins 2004; Iacono et al. 2018; Ostiguy et al. 2012; Serravalle et al. 2021). Indeed, the OBD, relative to control offspring, report more chronic interpersonal stress, which appears to be the familial instability created by parents with BD (Ostiguy et al. 2009). For these reasons, there has been increased attention to family-environmental factors in the study of the OBD.

Researchers have thus suggested that families should be the target of interventions for the OBD (Miklowitz et al. 2006). Studies have shown that family-focused therapy (FFT) is helpful for OBD between 9 and 17 years of age presenting with symptoms of BD, but not the full syndrome. That is, FFT was found to improve the course of BD in adulthood and even delay the onset of the disorder altogether (Miklowitz et al. 2006, 2011, 2014). More recently, a multisite randomized controlled trial (RCT) demonstrated that OBD who took part in FFT had longer intervals between recovery and onset of depressive episodes as compared to those who underwent 6 sessions of enhanced usual care (Miklowitz et al. 2020). These findings highlight the impact of FFT on improving the course of mood disorders post-treatment. However, to our knowledge, no psychosocial prevention efforts have targeted the OBD in middle childhood, prior to the manifestation of clinically significant symptoms of an affective disorder.

To address this gap in the literature, we have developed a family-based preventative intervention program titled Reducing Unwanted Stress in the Home (RUSH) to target families having unaffected 6–11 year-old OBD (i.e., who have not yet developed symptoms of an affective disorder). The RUSH program targets the stressful and chaotic family environment of the OBD, with a particular focus on improving organization, consistency, coping, and parenting practices, and aims to prevent the early development of internalizing and externalizing symptoms in OBD. The larger RUSH project was meant to be a proof-of-concept study in comparing OBD to offspring of parents with no affective disorders (i.e., control offspring). The RUSH intervention itself was not associated to direct reductions in symptoms in the OBD (Serravalle et al. in preparation). However, it has been suggested that the success of early interventions is dependent on actual changes in children’s environment (Sameroff and Fiese 2000). For example, positive changes in parent–child interactions in the OBD following the RUSH program were associated with a greater decline in internalizing problems relative to families where no improvement in parent–child interactions was observed (Serravalle et al. 2021). Thus, the current study explores whether RUSH-elicited changes in parenting stress, defined as stress stemming from one’s role as a parent, might lead to improved outcomes in the OBD.

Parenting stress is a unique type of stress, as evidenced by its adverse effects on parent–child interactions and on offspring emotional and behavioural functioning (Holly et al. 2019; Louie et al. 2017). In fact, some studies have shown a direct link between parenting stress and offspring behaviour problems (Neece et al. 2012; Verkleij et al. 2015). Some suggest that parenting stress may increase familial conflict and neglectful parenting practices, which may explain its detrimental effect on at-risk youths (Gerdes et al. 2007; Repetti et al. 2002). More specifically, parenting stress may mediate the relationship between affective disorders in parents and offspring development (Fredriksen et al. 2019). Given that parents with BD are known to experience acute levels of parenting stress (Jones et al. 2017), it may be a viable intervention target for families having a parent with BD.

Our first aim was to determine whether families having a parent with BD would report immediate (i.e., pre-post intervention) improvements in parenting stress and chronic interpersonal stress. Second, we investigated whether these reported improvements would be sustained over time (i.e., at three and 6-month follow-up). Third, we aimed to assess whether risk-status (i.e., OBD vs. control offspring) accounted for any variability in levels of stress prior to the start of the intervention, or rates of change in both types of stress across time. Last, we investigated whether improvements across time in parenting stress or chronic interpersonal stress reported by parents with BD mediated the association between participating in the RUSH program and offspring’s internalizing and externalizing symptoms across follow-up. In this proof-of-concept study, OBD were compared to control offspring—who completed all assessments but did not participate in the RUSH intervention. This allowed us to compare OBD with control offspring at baseline, and account for effects attributable to the passage of time or participating in a research project.

We hypothesized that families having a parent with BD would report significant post-intervention and long-term changes in parenting stress. We also hypothesized that there would be significant differences in the level of parenting stress and chronic interpersonal stress reported at baseline (i.e., intercepts) and the rates of change (i.e., slopes) between families having a parent with BD and control families. Relative to control families, families having a parent with BD were expected to report higher levels of both parenting and chronic interpersonal stress prior to the start of the intervention and follow significantly steeper trajectories of change over time. Lastly, we hypothesized that changes in parenting stress across all timepoints would mediate the relationship between participating in the RUSH program and internalizing and externalizing symptoms in OBD at follow-up.

## Methods

### Participants

Families were recruited through internet and newspaper services, local clinics, and patient support groups in Montréal, Québec. Families were mostly of white, middle-class, intact, and French-Canadian. Inclusion criteria for all families consisted of having at least one child between the ages of 6 and 11 years, and fluency in either English or French. General demographic information presented by risk status can be found in Table 1. Control families were excluded if either parent presented with a current axis-I disorder or reported a history of affective disorders. Inclusion criteria for families having a parent with BD consisted of have one parent with a BD1 or BD2 diagnosis. Psychopathology in parents was assessed with the Structured Clinical Diagnostic Interview for DSM-IV-R (SCID-I; 24). The sample consisted of 25 families with a parent having BD (72% mothers) and 28 families with parents having no mental disorders (90% mothers).

**Table 1:** Demographic characteristics presented by risk-status

Variable	OBD	Control Offspring
Offspring age at first timepoint	7.77 years (*SD* = 1.74)	8.67 years (*SD* = 1.68)
Offspring sex		
Girls	17	18
Boys	17	14
Family ethnicity		
Aboriginal (e.g., First Nations, Inuit, Metis, Native American, Native Australian)	1	0
Black (e.g., African–American, Nigerian, Haitian, Jamaican, Somali)	0	4
East Asian, South-East Asian, Pacific Islander (e.g., Chinese, Japanese, Korean, Vietnamese, Thai, Filipino, Indonesian)	1	2
Hispanic/Latino/Latin-American (e.g., Brazilian, Chilean, Mexican, Cuban)	1	3
Middle Eastern, North African, Central Asian (e.g., Jordanian, Saudi, Egyptian, Moroccan, Iranian, Afghan, Tajikistani)	2	3
White (Caucasian)	20	16
Parental marital status		
Single	5	2
Married	18	18
Separated	2	5
Divorced	0	3
Parental educational attainment		
Highschool Diploma	1	0
CÉGEP Diploma	4	4
Some university achievement	1	3
University Degree	19	21
Family annual income		
Less than $25,000	4	4
$25,001 to $50,000	8	8
$50,001 to $75,00	5	5
$75,001 to $100,000	1	7
More than $100,000	7	3
Family SES composite^a^	9.44 (*SD* = 2.10)	9.48 (*SD* = 1.67)

^a^SES Composite = socioeconomic composite score, which combines both parental educational attainment and family annual income

Within families having a parent with BD, most affected parents presented with BD-I (90%), and all reported a history of depression. At the start of the study, most parents with BD were asymptomatic, while two were in a current manic episode. While the latter two individuals were included in the study on the basis of their diagnosis, it was their partners who completed the RUSH program and all accompanying assessments. For the other 23 families, the affected parents attended the program and completed all assessments. All parents with BD were receiving pharmacological treatment at the time of the study, which included various combinations of antidepressant (bupropion, citalopram, escitalopram, sertraline, venlafaxine; n = 6), anticonvulsant (divalproex, lamotrigine, topiramate, valproate, n = 12), antipsychotic (chlorpromazine, lurasidone, olanzapine, quetiapine, ziprasidone; n = 12) and mood stabilizing medication (lithium; n = 9).

There were 66 children across the 53 families (34 OBD; 32 control; 48% female), aged between 6 and 11 years (*M* = 8.20 years, *SD* = 1.20 years). None of the control offspring met criteria for a psychological disorder, while ten OBD had a current diagnosis at T1, including an anxiety disorder (n = 1), enuresis (n = 2), oppositional defiant disorder (n = 1), and attention deficit/hyperactivity disorder (n = 6; all of whom were being treated with psychostimulants). None of the OBD were receiving any psychosocial treatments throughout the duration of the study. Psychopathology in offspring was assessed with the parent-version of the Kiddie-Schedule of Affective Disorders and Schizophrenia-Present and Lifetime Version [K-SADS-PL; (Kaufman and Schweder 2004). Children were excluded on the basis of presenting with pervasive developmental disorder, an intellectual or chronic physical disorder, or any history of an affective or psychotic disorder. Groups of children did not significantly differ on any key demographic variable (e.g., sex, ethnicity, or socioeconomic status) (all *p* > 0.05).

Of the initial 25 families having a parent with BD who underwent the T1 assessment, 20 completed the RUSH program. Of the 20 families who completed the RUSH program, all returned for T2 and T3 assessments, but only 17 families were retained at T4. Families most commonly reported a lack of time as the reason for dropping out at T4. No differences were observed between the original sample and those who dropped out prior to participating in the RUSH program or at T4 with regards to various demographic variables (offspring and parent sex and age, socioeconomic status), parental diagnosis (BD-I v. BD-II), offspring psychopathology at T1, as well as parents’ baseline scores across all four scores of parenting stress (all p > 0.05).

## Measures

### Parenting Stress Index, Fourth Edition Short Form (PSI-4 SF)

The PSI-4-SF (Abidin 2012) is a 36-item questionnaire aimed at evaluating various domains of stress related to parenting. The questions assess both parent and child characteristics which can exacerbate stress, as well as situational and demographic life stressors. This short form yields four scores. The *parental distress* subscale represents the extent to which parents feel conflicted and depressed in their role as a parent. The *dysfunctional interaction* subscale identifies whether parents feel satisfied of their child and the interactions they share with them. The *difficult child* subscale assesses the parent’s perception of their child, and whether they are difficult to take care of. Finally, the *total stress* score combines the latter scores to represent child characteristics (e.g., adaptability, demandingness, mood), parent characteristics (e.g., competence, isolation, attachment), and situational stressors. Higher scores represent more dysfunction. One parent from each family completed the questionnaire at each timepoint (85% mothers). The PSI-4-SF yields good psychometric properties, with moderate-to-excellent internal consistency for the four scales (*a* = 0.71–0.92) and adequate-to-strong reliability [*k* = 0.68–0.84; (Abidin 2012)].

### Behavior Assessment System for Children, Second Edition (BASC-2)

The BASC-2 assesses children’s internalizing (anxiety, depression, and somatic complaints) and externalizing (hyperactivity, aggression, and conduct problems) difficulties at home (Parent Rating Scales, PRS; 28). Higher scores on either scale represent more dysfunction. The BASC shows adequate test–retest reliability [k = 0.64–0.95; (Reynolds and Kamphaus 2002)] and high internal consistency [a = 0.80–0.90; (Merydith 2001)].

### UCLA Life Stress Interview

The UCLA semi-structured interview (Adrian and Hammen 1993; Hammen 1991) assesses the levels of both interpersonal and non-interpersonal stress in individuals’ lives over the last six months. Questions span nine life domains, and are scored using a 5-point scale, where higher scores represent higher levels of stress and more dysfunction. The interview may yield two subscale scores, differentiating between interpersonal and non-interpersonal stress; interpersonal stress is represented as the sum of scores across the domains of close friends, social life, romantic relationships, and family relationships, whereas non-interpersonal functioning consists of the domains of school, work, finances, health, and health of family members (Hammen et al. 2004; Eberhart and Hammen 2006). The score used in this study represents reported levels of chronic interpersonal stress, given the aforementioned hypotheses. Importantly, the UCLA provides an objective, observer rated representation of participants’ stress-related dysfunction.

### Intervention program: Reducing Unwanted Stress in the Home (RUSH)

The RUSH program aims to improve the quality of the caregiving environment and strengthen stress-coping and resilience among the OBD and their parents. This new prevention program was developed from validated cognitive-behavioural treatments for stress-coping, family relationships, child-rearing, and the management of bipolar disorder (Abramowitz 2012; Kendall and Hedtke 2006; Severe 2000; Shapiro and Sprague 2009). The program consists of 12 manual-based, closed weekly group sessions; parent and child sessions were run separately but simultaneously.

Parent sessions lasted two hours and were divided into three core modules: problem-solving skills, healthy communication, and organization and discipline in the home. Sessions also provided psycho-education about stress, its negative impact on families, and adaptive stress-coping strategies. Parents were provided with bi-weekly, 15-min booster calls aimed to encourage the use of skills in the home and provide individualized support. Child sessions lasted one hour, followed by an hour of play. Sessions were geared towards enhancing resilience while teaching age-appropriate coping strategies, cognitive restructuring, problem-solving, emotion labelling, relaxation, and assertiveness.

Therapist competence (child group: 5.65 ± 0.40; adult group: 5.44 ± 0.30, on a six point scale) and adherence to intervention protocol (child group: 2.88 ± 0.40; adult group: 2.81 ± 0.30, on a three point scale) were assessed by a trained observer who coded video recorded sessions. A second observer coded a random sample of videos (30%), and established good inter-rater reliability (ICC = 0.89–0.98). The coding scheme used by observers was a modified version of a previously validated scheme developed for cognitive-behavioural group treatments of adults (Hepner et al. 2011).

### Procedure

Parents first underwent a brief telephone interview to assess for eligibility. Next, parents were invited to the University to undergo a diagnostic interview (SCID-I). If eligible for the study, parents underwent the pre-intervention T1 assessment where they filled out questionnaires assessing parenting stress and child emotional and behavioural adjustment. Parents also underwent the UCLA Life Stress Interview, as well as a structured parent–child interaction task (Serravalle et al. 2021). The offspring underwent neuropsychological testing and provided saliva samples at home to assess cortisol levels (not reported here). The assessments were repeated at post-intervention (T2), as well as three (T3) and six (T4) months following the end of the RUSH program.

Following the T1 assessment, parents with BD were enrolled into the RUSH program, in groups of 3 to 10 participants. The number of sessions attended by families having a parent with BD varied between 8 and 12 (*M* = 11.15, *SD* = 1.18). Participants were remunerated for the assessments with CDN$100 at T1 and T4, and CDN$80 at T2 and T3. Children received small toys for their participation. Voluntary and informed consent and assent were obtained from the parents and their offspring, respectively, to participate in the study and have their data published. All procedures were approved by the Human Research Ethics Committee at Concordia University, Montréal, Canada (certification number: 30002475).

### Data analysis

The main analyses were conducted on SPSS version 28 (Corp 2021), with mixed effects modelling using maximum likelihood estimation (Heck et al. 2013). We first assessed the immediate improvements (pre-post intervention) reported by families having a parent with BD, followed by long-term changes reported across the four assessments. We then modeled changes in parenting stress and chronic interpersonal stress for the entire sample to investigate whether risk-group (OBD vs. controls) accounted for any variability in intercepts and slopes. Scores on parenting and chronic interpersonal stress were nested within time, and an auto-regressive heterogeneity covariance structure was specified. Offspring age was entered as a covariate for the analyses as to account for variability attributable to the 5-year age-range. Offspring sex was also entered as a covariate. All variables were standardized prior to running any statistical analyses.

**Fig. 1:**
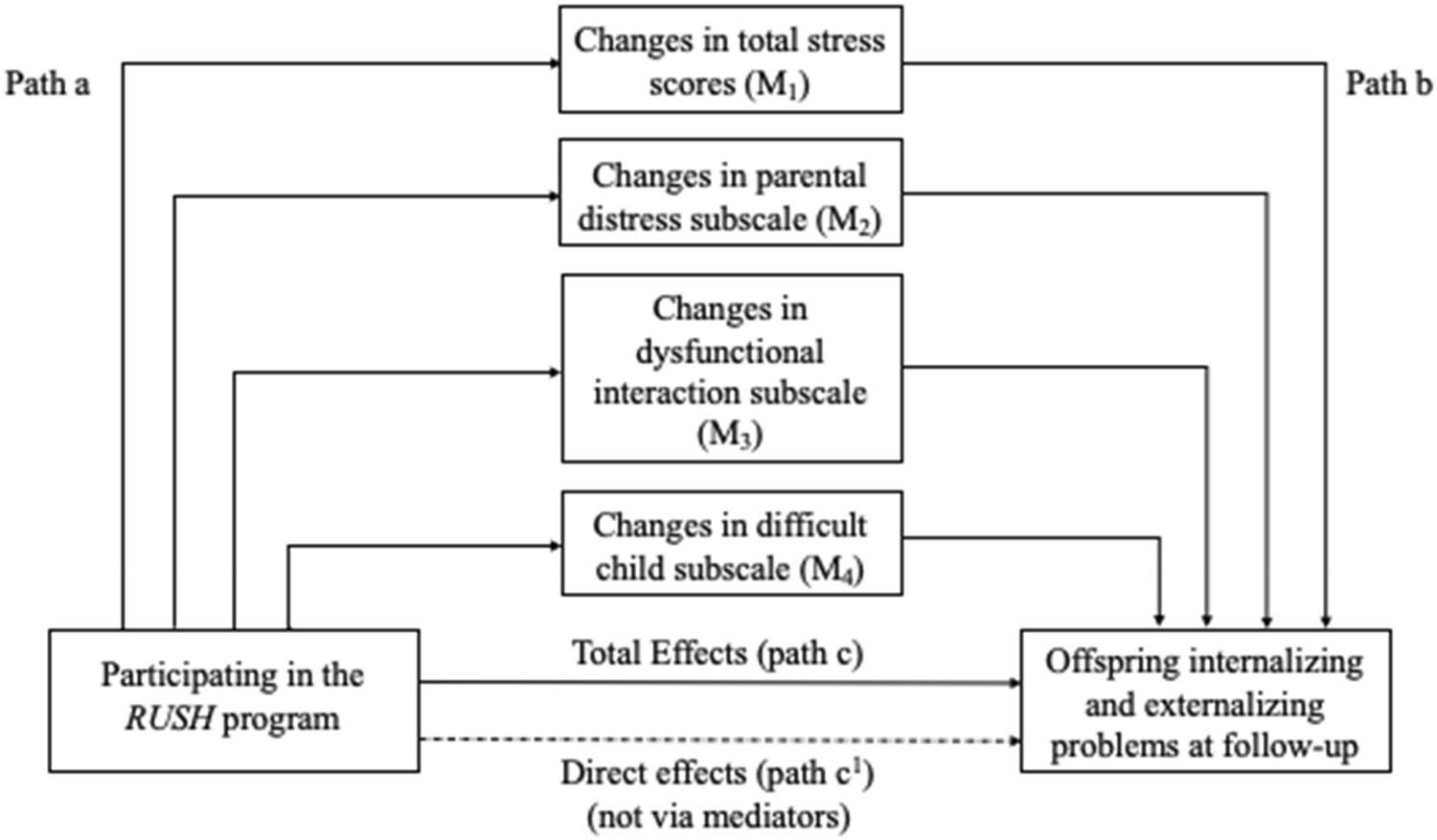
*Note:* Included mediators represent scores yielded from the Parenting Stress Index. Participating in the *RUSH* program is considered equivalent to having a parent with BD or not (OBD vs. control offspring)

Parallel mediations were run using Mplus version 8.0 (Muthén and Mplus 2017). These analyses aimed to determine whether intervention-related improvements in parenting stress or chronic interpersonal stress across time predicted offspring internalizing and externalizing symptoms at follow-up. A conceptual representation of this model can be seen in Fig. 1. Change scores were calculated by subtracting scores at T4 from scores obtained at T1. Greater positive change scores were indicative of greater reductions in parenting stress across time. There was no evidence that the data were not missing completely at random (MCAR), given Little’s MCAR test (Little 1988) (p = 1.00). For the three families having a parent with BD who completed T3 assessments but discontinued participation at T4, missing data were handled using full information maximum likelihood estimation. The strength of indirect effects are discussed using 95% bias corrected bootstrapped confidence intervals (MacKinnon et al. 2004; Miočević et al. 2018). The bootstrap sample was set to 5000 iterations. Means and standard deviations of reported parenting stress by families having a parent with BD and control families at each assessment phase can be seen in Table 2.

**Table 2:** Means and standard deviations for parenting stress across time and groups

	T1	T2	T3	T4
Variable	Mean (*SD*)	Mean (*SD*)	Mean (*SD*)	Mean (*SD*)
Total stress				
OBD	50.35 (18.71)	42.04 (24.61)	29.68 (13.75)	37.65 (23.54)
Control offspring	26.28 (10.94)	32.33 (18.79)	29.10 (16.23)	34.52 (23.01)
Parental distress				
OBD	18.71 (7.18)	16.08 (9.89)	14.16 (8.89)	4.65 (6.77)
Control offspring	8.41 (5.12)	11.07 (8.69)	12.73 (10.80)	4.72 (9.33)
Interaction				
OBD	12.09 (8.88)	9.60 (8.83)	9.20 (8.33)	8.40 (8.92)
Control offspring	4.78 (4.76)	7.03 (7.04)	8.57 (7.88)	7.72 (8.39)
Difficult child				
OBD	19.56 (6.25)	16.36 (7.81)	15.52 (6.46)	16.40 (7.82)
Control offspring	13.09 (3.84)	14.23 (5.37)	16.37 (6.42)	15.59 (6.21)

*OBD* offspring of parents with bipolar disorder, *T1* pre-intervention, *T2* post-intervention, *T3* 3-month follow-up, *T4* 6-month follow-up

## Results

### Changes in parenting and chronic interpersonal stress in families having a parent with BD

Families having a parent with BD reported significant pre-post improvements on the difficult child subscale (*b* = − 0.40, *SE* = 0.16, *p* = 0.016), but not on the parental distress subscale (*b* = − 0.25, *SE* = 0.20, *p* = 0.232), the dysfunctional interaction subscale (*b* = − 0.24, *SE* = 0.15, *p* = 0.132), or on the total stress score (*b* = − 0.32, *SE* = 0.18, *p* = 0.079). Reported pre-post changes in chronic interpersonal stress were not significant for families having a parent with BD (*b* = 0.10, *SE* = 0.22, *p* = 0.661).

**Fig. 2:**
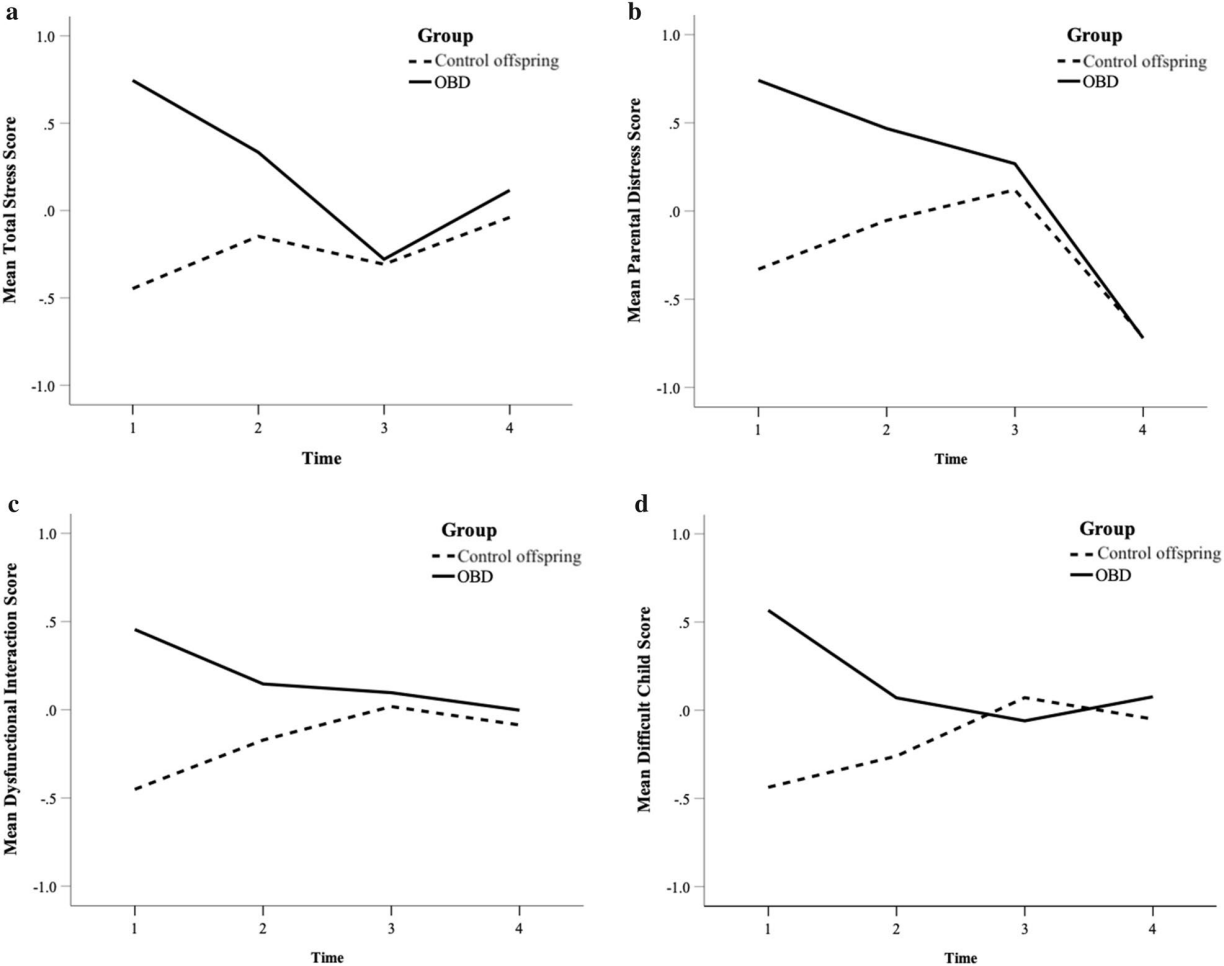
*Note:* The linear effect of time on all four subscales by intervention group. *OBD* offspring of parents with bipolar disorder

Across the four assessment points, reported changes on the difficult child subscale followed a quadratic curve (*b* = 0.12, *SE* = 0.04, *p* = 0.008). While improvements were noted until T3, scores on the difficult child subscale significantly increased at T4. Similarly, changes on the total stress score also followed a quadratic curve (*b* = 0.26, *SE* = 0.05, *p* < 0.001); scores improved until T3, but worsened at T4. Finally, changes on the parental distress subscale across time followed a linear curve (*b* = − 0.12, *SE* = 0.07, *p* < 0.001), with continued improvements reported until T4. Linear change on the dysfunctional interaction subscale also improved over the four timepoints, although the effect fell short of conventional levels of statistical significance (*b* = − 0.28, *SE* = 0.15, *p* = 0.063). In sum, despite a small post-intervention effect, improvements in parenting stress continued long after the termination of the intervention (see Fig. 2, panels a through d). In terms of chronic interpersonal stress, no significant changes were reported by families having a parent with BD across the four timepoints (*b* = 0.07, *SE* = 0.07, *p* = 0.319).

### Between‐group differences between families having a parent with BD and control families

#### Differences in reported levels of parenting and life stress at baseline

There was statistically significant variation in the intercepts of the total stress score (*Wald Z* = 4.07, *p* < 0.001), the parental distress subscale (*Wald Z* = 2.88, *p* = 0.004), the dysfunctional interaction subscale (*Wald Z* = 4.47, *p* < 0.001), and the difficult child subscale (*Wald Z* = 4.37, *p* < 0.001). In addition, there was significant variation in the intercept of the chronic interpersonal stress score (*Wald Z* = 4.11, *p* < 0.001). These findings indicate that between-subject effects influence scores at T1.

Risk-status significantly predicted intercepts for the total stress score (*b* = − 1.19, *SE* = 0.19, *p* < 0.001), as well as the parental distress (*b* = − 1.04, *SE* = 0.16, *p* < 0.001), dysfunctional interaction (*b* = − 0.91, *SE* = 0.22, *p* < 0.001), and difficult child subscales (*b* = − 0.99, *SE* = 0.20, *p* < 0.001). Significant intercept effects indicated that families having a parent with BD, as expected, reported higher levels of parenting stress than control families at T1. Additionally, risk status predicted initial levels of chronic interpersonal stress (*b* = − 0.54, *SE* = 0.27, *p* = 0.044). Neither offspring sex or age at T1 accounted for any variability in intercepts.

### Differences in reported changes in parenting and chronic interpersonal stress over time

There was statistically significant variability in the linear effects of time for the difficult child subscale (*Wald Z* = 2.27, *p* = 0.023), which indicates that between-subject effects influence its rate of change. For the total stress score (*Wald Z* = 1.77, *p* = 0.076) and the dysfunctional interaction subscale (*Wald Z* = 1.86, *p* = 0.063), the amount of inter-individual variability was trend-level. Finally, for the parental distress subscale, the observed between-subject differences in changes over time were not statistically significant (*Wald Z* = 1.34, *p* = 0.180). In terms of chronic interpersonal stress, the level of variability in the linear effects of time was non-significant (*Wald Z* = − 0.723, *p* = 0.470).

Change across time varied by risk-status for the total stress score (*b* = 0.95, *SE* = 0.18, *p* < 0.001), the dysfunctional interaction subscale (*b* = 0.64, *SE* = 0.21, *p* = 0.003), and for the difficult child subscale (*b* = 0.94, *SE* = 0.23, *p* < 0.001) (see Fig. 1a, c, d, respectively). Families having a parent with BD reported changes which followed a steeper curve than control families. Upon further investigation, families having a parent with BD also reported greater pre-post improvement on both the total stress score (*b* = 0.54, *SE* = 0.24, *p* = 0.027) and on the difficult child subscale (*b* = 0.56, *SE* = 0.22, *p* = 0.012). Finally, the rate of change for chronic interpersonal stress scores did not vary between groups (*b* = − 0.01, *SE* = 0.09, *p* = 0.925); this means that improvements in observer-rated levels of stress were not associated to risk-status. Offspring sex and age were not significantly associated to variability in growth trajectories.

### Parallel mediations models

Standardized model results predicting both internalizing and externalizing symptoms are summarized in Tables 3 and 4, respectively. Internalizing and externalizing scores were each averaged across T3 and T4. The mean internalizing score (± *SD*) for OBD was 12.64 (11.71), while it was 9.11 (8.10) for control offspring. The mean externalizing score for OBD was 17.08 (15.36), and 6.72 (8.72) for control families. Mediations were run using change scores on parenting stress, however changes in chronic interpersonal stress were omitted from these analyses given the non-significant slope terms for families having a parent with BD reported above.

**Table 3:** Standardized model results of parallel mediations predicting parent-reported internalizing problems across follow-up

Mediator	Effect of *IV* on mediator (*a*)	Unique effect of mediator (*b*)	Indirect effect (*ab*)	BC 95% CI
	β (SE)	β (SE)	β (SE)	
Total stress	0.41 (0.10)**	− 0.75 (0.19)**	− 0.31 (0.10)**	[− 0.55, − 0.13]
Parental distress	0.38 (0.12)*	0.16 (0.16)	0.07 (0.07)	[− 0.05, 0.22]
Interaction	0.28 (0.12)*	0.30 (0.19)	0.07 (0.08)	[− 0.07, 0.22]
Difficult child	0.32 (0.13)*	0.11 (0.15)	0.01 (0.05)	[− 0.07, 0.14]
Total indirect	–	–	− 0.15 (0.06)*	[− 0.31, − 0.03]

*IV *independent variable, *BC *bias-corrected bootstrap, *CI *confidence interval

^*^p < .05, **p < .001

^a^The (*a*) paths are identical across both mediation models

**Table 4:** Standardized model results of parallel mediations predicting parent-reported externalizing problems across follow-up

Mediator	Effect of *IV* on mediator (*a*)	Unique effect of mediator (*b*)	Indirect effect (*ab*)	BC 95% CI
	β (SE)	β (SE)	β (SE)	
Total stress	0.41 (0.10)**	− 0.84 (0.09)**	− 0.35 (0.10)**	[− 0.57, − 0.16]
Parental distress	0.38 (0.12)*	0.14 (0.15)	0.07 (0.07)	[− 0.05, 0.23]
Interaction	0.28 (0.12)*	0.42 (0.14)*	0.12 (0.06)	[0.01, 0.26]
Difficult child	0.32 (0.13)*	0.18 (0.11)	0.06 (0.05)	[0.00, 0.21]
Total indirect	–	–	− 0.10 (0.06)	[− 0.22, 0.02]

*IV* independent variable, *BC* bias-corrected bootstrap, *CI *confidence interval

^*^p < 0.05, **p < 0.001

^a^The (*a*) paths are identical across both mediation models

Improvements on the total stress subscale mediated the relationship between participation in the RUSH intervention program and internalizing symptoms across follow-up (β = − 0.31, SE = 0.10, Cl = − 0.55, − 0.13; Table 2). Additionally, reported improvements on the total stress subscale also mediated the relationship between participation in the RUSH program and externalizing symptoms across follow-up (β = − 0.35, SE = 0.10, Cl = − 0.57, − 0.16; Table 3). None of the other indirect effects were significant.

## Discussion

Three key findings emerged from the present study. First, families having a parent with BD reported more parenting stress than control families pre-intervention. Families having a parent with BD reported less perceived difficulty in caring for their child (i.e., difficult child subscale), as well as less overall interpersonal and situational stress (i.e., total stress score) immediately following the end of the RUSH intervention than control families, and continued to do so until 6-months post-intervention. Second, the relationship between participating in the RUSH program and OBD’s internalizing and externalizing problems at follow-up was mediated by decreased parenting stress, as assessed via the total stress score. Third, families having a parent with BD also reported more chronic interpersonal stress than control families pre-intervention. However, these families did not report significant changes across time on chronic interpersonal stress. Interestingly, the RUSH program had robust effects in reducing aspects of parenting stress over time but had no impact on chronic interpersonal stress. Thus, the intervention was particularly helpful in reducing perceptions of stress around one’s role as a parent, but did not influence interpersonal stress more generally.

Parents living with affective disorders experience detrimental levels of parenting stress (Jones et al. 2017; Gelfand et al. 1992), and studies have shown that parenting stress may mediate the relationship between parents’ depressive symptoms and offspring developmental outcomes (Fredriksen et al. 2019). Thus, the relationship between parenting stress and offspring development is especially relevant in the study of offspring of parents with affective disorders. In line with the current findings, Jones et al. (Jones et al. 2017) reported that an online parenting intervention for families having a parent with BD led to significant improvements in both parenting stress and offspring emotional and behavioural problems. However, the present study is the first to demonstrate that the relationship between participating in a preventative intervention and OBD internalizing and externalizing symptoms is mediated by improvements in parenting stress. In addition, the finding that families with a parent having BD have higher parenting stress than control families is an important addition to the literature; only one study thus far had demonstrated differences on the Parenting Stress Index between depressed and non-depressed mothers (Gelfand et al. 1992). This finding complements the previously documented dysfunction present in the family environment of parents with BD (Iacono et al. 2018; Ostiguy et al. 2012; Serravalle et al. 2021).

Support for our hypotheses was mixed. Pre-post intervention changes in parenting stress were found only on the difficult child subscale. However, there were marked long-term improvements for three of the four parenting stress subscales (i.e., the total stress, parental distress, and difficult child subscales). This trend is similar to the findings from Jones and colleagues (Jones et al. 2017), where larger improvements in parenting stress were reported throughout follow-ups, but not immediately post-intervention. Additionally, changes across time for both the total stress and difficult child subscales were not stable. Following steady improvements, levels of reported parenting stress had increased significantly by the final timepoint. However, nonlinear trajectories of change are common following clinical interventions; many individuals reporting initial improvements will eventually stabilize, or sometimes even worsen before improving again (Owen et al. 2015). Importantly, levels of parenting stress reported by families having a parent with BD were comparable to those of control families at the final timepoint.

The present results highlight the value of targeting parenting stress when working with at-risk families. The results present early evidence in favour of preventative approaches for at-risk youths. Intervening in middle childhood, prior to the onset of affective disorders in adolescence (Warner et al. 1992), may be central to effective prevention efforts. Without the urgent need to manage offspring symptoms, such as interventions catered to an older population (Compas et al. 2009; Garber et al. 2009), the RUSH program can target well-established family-related risk factors for OBD (Serravalle et al. 2021). Despite the positive implications of our findings, this study was not without limitations. The sample size was small, limiting statistical power. However, mixed effects modelling with maximum likelihood estimation was used to minimize this limitation. The sample consisted of mostly white French-Canadians which may limit the generalizability of our findings. Finally, the present study did not utilize a RCT design—the gold standard in intervention research. In this proof-of-concept project, the control families served only as a comparison group. While our results provide evidence that families having a parent with BD benefitted from the RUSH intervention, we are unable to conclude if the RUSH intervention would fare better than a waitlist or active control intervention in families having a parent with BD. However, the effects reported here support the need for future RCTs to explore the efficacy of the RUSH program, as well as putative mechanisms of change.

To conclude, the present findings contribute to the literature on the role of the caregiving environment in the development of OBD (Iacono et al. 2018). This study presents findings that highlight the link between reported improvements on parenting stress following the RUSH program and OBD functioning. Our findings build on recent developments in family-based approaches to the treatment of BD (Miklowitz and Chung 2016). Broader implications may lie in adopting a preventative approach for at-risk youths such as OBD. Future studies should submit the RUSH intervention to a more rigorous RCT design to better understand its benefits as compared to other types of interventions, and for other at-risk populations.

## Data Availability

The datasets generated and/or analysed during the current study are not publicly available but are available from the corresponding author upon request.

## Abbreviations

OBD: Offspring of parents with bipolar disorder
BD: Bipolar disorder
RUSH: Reducing Unwanted Stress in the Home
FFT: Family-focused therapy
RCT: Randomized controlled trial
